# Some Problems and Our Steps to Solve Them

**Published:** 1921-02-05

**Authors:** 


### RECENT TUBERCULOSIS WORK.

### Some Problems and Our Steps to Solve Them.

Lupus seems to stand apart from other tuberculous diseases. Bacilli isolated from lupoid lesions are of uncommonly low virulence. In course, it is far milder and more chronic than tubercle shows itself elsewhere. With, say, pulmonary phthisis lupus has a kind of inverse ratio: it has been known to heal completely and suddenly, after many years, with the onset of lung complication, and to get worse synchronously with the arrest of consumption. It is much more confined in geographical range than the other forms of tuberculosis, being rare in hot countries. It seems to form an exception to Cornet's law of regional involvement of lymphatic glands: the nearest glands may caseate, "but," says Treves, "this, considering the true nature of lupus, is surprisingly rare." And, finally, it is much less associated with depression of the general health than one looks for in tuberculosis. This last point receives confirmation from statistics given in a recent number of *L'Attualità Medica*, representing war-time mortality experience. Deaths from practically every other form of tubercle rose during the war years, as compared with those from 1911 to 1914, in the way with which we are unhappily familiar. But in the case of lupus they did not. Of course, the disease is not commonly a fatal one, but deaths from it do occur; there were, in 1911, as many as fifty-six in England and Wales alone.

### An Unusual Instance.

Kornitzer, in the *Berliner kliniker Wochenschrift*, gives a case that must be nearly unique; and that would have baffled a good many who might have prided themselves on their powers of early diagnosis of phthisis. A man forty years old, after seventeen months' service in the field, fell ill with signs and symptoms of chronic tuberculous pleurisy. The case ended unfavourably, and the *post-mortem* revealed the following complex and disconcerting findings. There was a right-sided hæmorrhagic pleurisy, with an endothelioma of the right pleura and metastatic deposits in the diaphragm, and in the thoracic and abdominal lymphatic glands. In the left pleura was found a most curious neoplasm of another kind. It was a fibroleiomyoma the size of an apple, taking origin from the posterior surface of the left lung, and projecting into the pleural space on a long slender fibrous peduncle; the whole neoplasm being covered with pleural endothelium continuous with that of the visceral pleura. There was, too, in the tumour of the left side actually a microscopically small metastasis from the endothelioma in the opposite half of the chest. The case is further remarkable in that a myomatous tumour has not, according to the author, been previously described as affecting the pleura. They develop as a rule, of course, from non-striated muscular fibres. Hence he supposes it arose in this case from the sub-pleural tissue.

### Border-line Cases.

It has been known for a fairly long time that between Hodgkin's disease and tuberculosis of the lymphatic glands there may almost be made out a connecting chain of borderline cases. The name of lymphogranulomatosis has been given to some such. Gebsattel has recently described an atypical case of multiple glandular enlargement, without caseation, but accompanied by a large, hard, uniform splenic tumour, and also slight change in the bone marrow. The autopsy investigation showed no tubercle bacilli in any organ, although giant cells were present. Inoculation of material from the spleen proved tuberculosis, however, since a guinea-pig killed after the lapse of seven weeks yielded fresh caseous tubercle of the spleen and liver, while the bacillus of Koch was present in sections of all its organs. This case shows that the differential diagnosis, during life, between Hodgkin's disease and scrofula may be uncommonly difficult. The blood picture may fail to be of any help, and in the absence of caseation a proliferative, hyperplastic lymphgland tuberculosis can simulate leukæmia with great faithfulness.

### Chemio-Therapy in Tuberculosis.

Copper preparations have now an acknowledged sphere of usefulness in, at any rate, skin tuberculosis. It is therefore of interest to note that Ehrlich's old pupil, Countess von Linden, who has continued Finkler's original work on the chemotherapy of tuberculosis, claims to have immunised rabbits to a large extent against the disease by previous intravenous or oral administration of copper preparations. This partial immunity may persist for as long as nine months, and she contrasts it with the transitory protection (three weeks only) found by Hata to be afforded by salvarsan against spirillosis. In the case of rabbits and tuberculosis, immunity is shown in a chronic isolated, instead of a rapid and general, infection, with the escape of organs usually especially vulnerable, like the kidneys, and with the normal course of gestation in infected animals, as against early miscarriage. Protection is also afforded against "natural" infection, as by contact of young rabbits with badly infected mothers. Animals, as notoriously susceptible as guinea-pigs even, have been shown to be favourably affected, when already tuberculous, by the administration of copper, and such treatment in those with greater natural resistance has resulted in cure.

There is no disease, the pathology of which appears, at one time, to be so well understood, and yet, at another, to present so many small but puzzling inconsistencies. Daily, however, these problems seem to come one step nearer to their solution, and in the interests of humanity no scientific effort could be directed more wisely.

